# Effect of mutations to amino acid A301 and F361 in thermostability and catalytic activity of the β-galactosidase from *Bacillus subtilis* VTCC-DVN-12-01

**DOI:** 10.1186/s12858-016-0070-0

**Published:** 2016-07-08

**Authors:** Thao Thi Nguyen, Hanh Van Vu, Nhung Thi Hong Nguyen, Tuyen Thi Do, Thanh Sy Le Nguyen

**Affiliations:** Institute of Biotechnology, Vietnam Academy of Science and Technology, 18 Hoang Quoc Viet Road, Distr. Caugiay, 10600 Hanoi, Vietnam

**Keywords:** β-Galactosidase, Mutants, *Bacillus subtilis*, *Escherichia coli*, Error prone rolling circle amplification, Catalytic activity

## Abstract

**Background:**

Beta-galactosidase (EC 3.2.1.23), a commercially important enzyme, catalyses the hydrolysis of β-1,3- and β-1,4-galactosyl bonds of polymer or oligosaccharidesas well as transglycosylation of β-galactopyranosides. Due to catalytic properties; β-galactosidase might be useful in the milk industry to hydrolyze lactose and produce prebiotic GOS. The purpose of this study is to characterize β-galactosidase mutants from *B. subtilis*.

**Results:**

Using error prone rolling circle amplification (epRCA) to characterize some random mutants of the β-galactosidase (LacA) from *B. subtilis*VTCC-DVN-12-01, amino acid A301 and F361 has been demonstrated significantly effect on hydrolysis activity of LacA. Mutants A301V and F361Y had markedly reduced hydrolysis activity to 23.69 and 43.22 %, respectively. Mutants the site-saturation of A301 reduced catalysis efficiency of LacA to 20–50 %, while the substitution of F361 by difference amino acids (except tyrosine) lost all of enzymatic activity, indicating that A301 and F361 are important for the catalytic function. Interestingly, the mutant F361Y exhibited enhanced significantly thermostability of enzyme at 45–50 °C. At 45 °C, LacA-361Y retained over 93 % of its original activity for 48 h of incubation, whereas LacA-WT and LacA-301Vwere lost completely after 12 and 24 h of incubation, respectively. The half-life times of LacA-361Y and LacA-301 V were about 26.8 and 2.4 times higher, respectively, in comparison to the half-life time of LacA-WT. At temperature optimum 50 °C, LacA-361Y shows more stable than LacA-WT and LacA-301 V, retaining 79.88 % of its original activities after 2 h of incubation, while the LacA-WT and LacA-301 V lost all essential activities. The half-life time of LacA-361Y was higher 12.7 and 9.39 times than that of LacA-WT and LacA-301 V, respectively. LacA-WT and mutant enzymes were stability at pH 5–9, retained over 90 % activity for 72 h of incubation at 30 °C. However, LacA-WT showed a little bit more stability than LacA-301 V and LacA-361Y at pH 4.

**Conclusions:**

Our findings demonstrated that the amino acids A301V and F361 play important role in hydrolysis activity of β -galactosidase from *B. subtilis*. Specially, amino acid F361 had noteworthy effect on both catalytic and thermostability of LacA enzyme, suggesting that F361 is responsible for functional requirement of the GH42 family.

## Background

β-Galactosidase (β-D-galactoside galactohydrolase, E.C 3.2.1.23), a commercially important enzyme, catalyses the hydrolysis of β-D-galactoside linkage in polymers, oligosaccharides, or other secondary metabolic products [1]. Some β-galactosidases may have an activity of transferring one or more D-galactosyl units onto lactose [2]. Due to this property, β-galactosidase have two main applications containing the removal of lactose from milk products for lactose intolerant people and production of galactosylated products [3–5]. β-galactosidase is one of the most popular technologies to produce lactose reduced milk and related dairy products for consumption by lactose intolerant people. β-Galactosidase is widely used to improve sweetness, solubility, flavor and digestibility of dairy products [6, 7]. Besides, β-galactosidase shows a high transgalactosylation activity, so that they are used for the synthesis of prebiotic galacto-oligosaccharides [8], novel galactosides [9]. The β-galactosidase activity also contributes to glycoprotein degradation [10], the degradation of GM1 ganglioside and other glycolipids and glycoproteins with a terminal galactose moiety [11].

β-Galactosidases are widely distributed in nature and produced by microorganisms (yeasts, fungi, bacteria, and archaea), plants [12, 13], and animals [14, 15]. At present, based on their sequence similarity and reaction mechanisms, β-galactosidase are classified into four main glycoside hydrolase families (GHFs), GHF-1, GHF-2, GHF-35 and GHF-42 [16]. The catalytic residues of these enzyme group, which are located at the β-4 and β-7 of the triose phosphate isomerase (TIM) barrel fold, are a member of the 4/7-superfamily with a TIM container fold catalytic domain. In general, β-galactosidases of GHF-1 and GHF-2 are found in mesophiles and demonstrate lactase activity, while enzymes belonging to families GHF-35 and GHF-42 are usually found in thermophiles and preferentially degrade β-1,4-linkages between two galactose moieties. However, lactose hydrolysis activity of GHF-35 and GHF-42 are absent or weak [17, 18].

Nowadays, to improve the efficiency of using thermophilic β-galactosidases, different strategies have been used, including screening enzymes from different species of *Bacillus*, cloning and expressing in a heterologous host system [19–22], reconstruction of the enzyme by protein engineering to create enzymes with novel properties. Among them, protein engineering was an efficient way. Recently, efforts have been made to alter substrate specificity, stability and specific activity of β-galactosidases belong to GHF-42 [23–26].

In a previous study, we cloned a gene (*lacA*) coding for β-galactosidase of GHF-42 from *Bacillus subtilis* strain VTCC-DVN-12-01, expressed in *E. coli* and the β-galactosidase LacA was purified and characterized [20]. This β-galactosidase has a potential application in food industry. There was no report on random mutagenesis of the β-galactosidase using epRCA. For the first time, we used epRCA to characterize β-galactosidase mutants from *B. subtilis*.

## Results

### Library construction and prescreening for β-galactosidase activity of transformants

Plasmid pELacA was amplified by the rolling circle mechanism in the presence of manganese ions, which has been shown to reduce the fidelity of DNA polymerase and cause random mutagenesis during RCA. The results of electrophoresis on agarose gel indicated that the most of epRCA products had a size range of more than 10,000 bp, which was multimeric forms of two or more repeated sequences of pELacA (7500 bp, monomeric form). The epRCA products were directly transformed into *E. coli* JM109(DE3), resulting in colonies containing a randomly mutated plasmid library.

However, a few transformants (10–15 transformants) per 18 μg of epRCA resulted when the epRCA products directly transformed into *E. coli* JM109(DE3). Interestingly, the epRCA products was digested with a single-cut restriction enzyme *Mlu*I followed by self-ligation by treatment with T4 DNA ligase which dramatically increased the transformation efficiency. As a result, approximately 700 transformants were obtained from 72 ng of epRCA supplemented with 1.5 mM of manganese chloride, the transformation efficiency was 116 folds higher than that of direct transformation of the epRCA products without digestion and ligation.

Transformants containing putative mutants in the *lacA* gene were grown in LB medium in deep well microplates for the β-galactosidase production. The whole lysates were used as enzyme sources to determine the LacA activity in 96-well microplates using oNPG as a substrate. In total 10,000 transformants were screened hydrolysis activity of mutant library, the results have been shown that most transformants (75 %) were no significant change in hydrolysis activity in comparison with the wild-type. About 20 % of transformants were a complete loss of the activity and about 5 % of transformants strongly decreased in the hydrolysis activity. There were only 0.03 % of transformants with higher hydrolytic activity than wild-type.

### Mutation analysis

Among total 10,000 transformants obtained from the libraries, the transformants epRCA125, epRCA221, and epRCA887 (higher 1.5–2 times LacA activity than the wild-type by prescreening), epRCA259 and epRCA461 (≤50 % LacA activity than the wild-type) were randomly selected for DNA sequence analysis of the *lacA* gene.

The transformants epRCA259 and epRCA461 showed changes in amino acids, but epRCA125, epRCA221 and epRCA887 did not (Table 1). The mutation F361Y and A524T in LacA259 and mutation E62V, R77W, A191V, and A301V in LacA461 led to decrease in enzyme activity by nearly 50 % and 80 %, respectively.

**Table 1:** Mutations of selected epRCA transformants

Transformant	RA (%)	Nucleotide substitution	Amino acid substitution
Wild-type	100	original	
LacA125	167	No	
LacA221	150	No	
LacA887	198	ACG → ACA, CGG → CGA	T408, R624
LacA259	52	UUC → UAU, GCU → ACU	F361Y, A524T
LacA461	36	GAG → GAA, CGG → UGG, GCG → GUG, GCG → GUG	E60, R77W, A191V, A301V

Plasmids from selected colonies were isolated and sequenced to identify mutations in the *lacA* gene

**Fig. 1:**
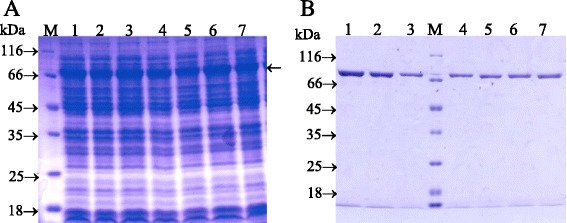
SDS-PAGE analysis of the level expression (A) and puried mutant proteins (B) of wild-type and mutants LacA. **a**. LacA-WT and mutants were expressed to homogeneity. Then total protein of cells were checked on SDS-PAGE to analysis the expression of LacA-WT and mutants. **b**. LacA-WT and mutants were puried to homogeneity using nickel chelate affinity chromatography. Homogeneous fractions were collected to analysis on SDS-PAGE. The protein samples were run on a 12.5 % SDS reducing gel and stained with Coomassie Briliant Blue R250. Lane 1–7: WT, E62V, R77W, A191V, A301V, F361Y and A524T, respectively; lane M: molecular marker

However, to give a clearer explanation of effecting of the mutations of E62V, R77W, A191V, A301V, F361Y and A524T on decrease of enzyme activity, we made point mutations to confirm. The wild-type WT and mutants F361Y, A524T, E62V, R77W, A191V, A301V were cultivated to express the recombinant β-galactosidase in *E. coli* JM109(DE3). SDS polyacrylamide gel electrophoresis analysis showed the expression level of wild-type was similar to mutant enzymes (Fig. 1a). The expression level obtained 18,86–19,27 % of total protein of cells, that was estimated by using the Dolphin-1D software. This indicated that the mutations did not affect the expression of LacA variants in comparison to the wild-type.

The LacA-WT and mutants LacA-E62V, LacA-R77W, LacA-A191V, LacA-A301V, LacA-F361Y and LacA-A524T were purified to homogeneity using nickel chelate affinity chromatography and showed a single protein band corresponding to the molecular mass of 70 kDa of the wild type LacA (Fig. 1b). The purification factor and yield of variants were similar to that of LacA-WT. The purification factor and yield obtained 4.08–4.98 and 71–84.1 %, respectively (Table 2). The specific activities of mutants were determined with oNPG substrate and compared with that of the wild-type enzyme (Table 3). The specific activity of mutants LacA-A524T, LacA-E62V, LacA-R77W and LacA-A191V did not show any significant changes, whereas LacA-A301V and LacA-F361Y remained the relative specific activity 23.69 and 43.22 %, respectively, in comparison with LacA-WT. These results indicated mutations F361Y of epRCA259 and A301V of epRCA461 decreased in enzyme activity. This suggested that A301 and F361 could play a significant role maintaining activity of LacA.

**Table 2:** Purification steps of LacA-WT and mutants from JM109(DE3)

Mutant LacA	Steps of purification	Total activity (U)	Total protein (mg)	Specific activity (U/mg)	Purification factor	Yield (%)
WT	Crude β-galactosidase	5.13	9.04	0.57	1.0	100
	Probond™resin	4.12	1.66	2.48	4.53	80.49
E62V	Crude β-galactosidase	4.53	8.18	0.55	1.0	100
	Probond™resin	3.81	1.59	2.39	4.35	84.1
R77W	Crude β-galactosidase	5.02	9.25	0.54	1.0	100
	Probond™resin	3.56	1.39	2.56	4.74	71
A191V	Crude β-galactosidase	4.32	8.72	0.50	1.0	100
	Probond™resin	3.54	1.42	2.49	4.98	81.94
A301V	Crude β-galactosidase	1.15	8.66	0.13	1.0	100
	Probond™resin	0.89	1.51	0.59	4.54	77.1
F361Y	Crude β-galactosidase	2.06	9.11	0.24	1.0	100
	Probond™resin	1.72	1.55	1.11	4.63	83.5
A524T	Crude β-galactosidase	5.11	8.25	0.62	1.0	100
	Probond™resin	4.1	1.62	2.53	4.08	80.23

Wild-type and mutants of LacA were purified to homogeneityby affinity chromatography Ni^2+^ ProBond™ resin. The relative activity of β-galactosidase was determined in 100 mM buffer Z (pH 7.0) at 55 °C with substrate oNPG (4 mg/ml)

**Table 3:** Specific activities of the wild-type and mutant LacA enzymes using oNPG as substrate

Transformant	Mutant LacA	Specific activity (U/mg)	Specific activity relative to wild-type (%)
WT		2.48 ± 0.06	100
259	F361Y	1.11 ± 0.075	44.42 ± 3.01
	A524T	2.53 ± 0.078	102.19 ± 3.16
461	E62V	2.39 ± 0.045	96.31 ± 1.8
	R77W	2.56 ± 0.068	103.39 ± 2.74
	A191V	2.49 ± 0.073	100.6 ± 2.95
	A301V	0.59 ± 0.014	23.69 ± 0.56

The relative activity of β-galactosidase was determined in 100 mM buffer Z (pH 7.0) at 55°Cwith substrate oNPG (4 mg/ml)

### Site-saturation mutagenesis

Saturation mutagenesis at position 301 and 361 was carried out to give a deeper understanding of the role of A301 and F361 in hydrolysis activity of LacA. About 1,000–1,200 colonies of each mutant library were tested their hydrolysis activity in 96-well plates with oNPG substrate. The result of screening activity of colonies in mutants library of F361X showed that 94.4 % of colonies in this library completely lost hydrolysis activity, 5.3 % of colonies had hydrolysis activity similar to wild-type, and three colonies was a decrease in activity from wild-type of between 30 and 50 %. Interestingly, the analysis result of *lacA* gene from all three colonies decreased activity showed Phe361 was replaced with tyrosine. Whereas, sequencing of three *lacA* genes been randomly selected from colonies had activity similar to wild-type showed no change amino acid at this position. In addition, three *lacA* genes from colonies without hydrolysis activity were sequenced, and we found that Phe361 was replaced with valine, cysteine and leucine. Again, these results demonstrate role of Phe361 of LacA was important in substrate recognition. Any changes in amino acid residues of this position may disturb the hydrogen bond network to induce a loss or decrease in hydrolysis activity of LacA.

In contrast to F361 position, the result of screening activity of colonies in library of A301X indicated that 83.2 % selected colonies were decreased out of 1,000 (compare to the wild-type colonies the decreased activities ranged 20–40 %), 6.4 % of colonies had hydrolysis activity similar to wild-type, whereas 10.4 % had no activity. We determined the changes A301E and A301Y of LacA from some colonies decreased activity. This result demonstrated that any change in position A301 did not affect to hydrolysis activity of LacA as strong as that of F361 position. Amino acid A301 might not bind to substrate in hydrolysis but affect to structure of enzyme.

### pH and temperature dependency of mutant enzymes

The specific activity of LacA-361Y and LacA-301 V decreased dramatically to 43.09 and 23.7 % in comparison with the specific activity of the LacA-WT, respectively (Table 5). Both mutants remained the relative specific activity 44.8 % and 22.6 % in comparison with LacA-WT, when they were expressed using the initial vector pET22b + .

**Fig. 2:**
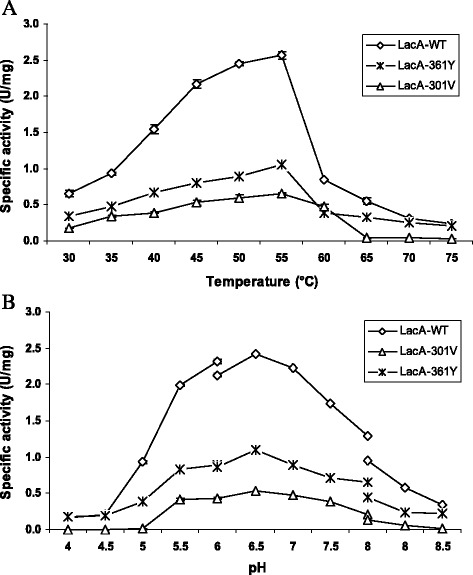
Temperature (**a**) and pH optimum (**b**) of Lac-WT, Lac-301 V and LacA-361Y. β-Galactosidase activity toward oNPG was determined in 100 mM buffer Z (pH 7.0). The pH was determined in different buffers by varying pH values from 4.0 to 9.0 at 55 °C using substrate oNPG (4 mg/ml). Na-acetate buffer pH 4–6, Na-phosphate buffer pH 6.0–8.0, Tris–HCl buffer pH 8.0–9.0

The optimum temperature and pH of LacA-WT, LacA-361Y and LacA-301 V were obtained at the same 50–55 °C and pH 6.5 (Fig. 2). However, mutants of A301V and F361Y decreased the specific activity from 2.57 ± 0.05 U/mg (100 %) of the LacA-WT to 0.66 ± 0.01 U/mg (26 %) and 1.05 ± 0.03 U/mg (41 %), respectively, at 55 °C (Fig. 2a). At the optimum pH, the specific activity also decreased from 2.42 ± 0.13 (100 %) for LacA-WT to 0.54 ± 0.001 U/mg (22 %) and 1.1 ± 0.008 (45 %), respectively (Fig. 2b). Thus, mutant points A301V and F361Y were not effects on optimum temperature and pH of LacA.

**Fig. 3:**
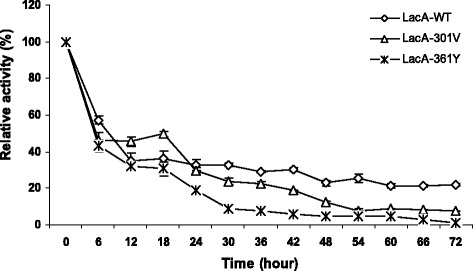
pH stability of Lac-WT, Lac-301 V and LacA-361Y. The purified LacA-WT, Lac-301 V and LacA-361Y were incubated in 100 mM Na-phosphate buffer pH 4. The relative activity of β-galactosidase toward oNPG (4 mg/ml) was determined in 100 mM buffer Z (pH 7.0) at 55 °C

The pH stability of LacA-WT, LacA-301 V and LacA-361Y remained most the same at pH 5.0 to pH 9.0. The hydrolytic activity of LacA-WT and mutant enzymes retained over 90 % at pH 5.0 to 9.0 after 72 h of incubation. However, LacA-WT showed a little more stability than LacA-361Y and LacA-301 V at pH 4 (Fig. 3). The hydrolytic activity of Lac-WT retained 24 % for 72 h of incubation at 30 °C, whereas LacA-301 V and LacA-361Y did not have activity after 42 and 60 h, respectively.

**Fig. 4:**
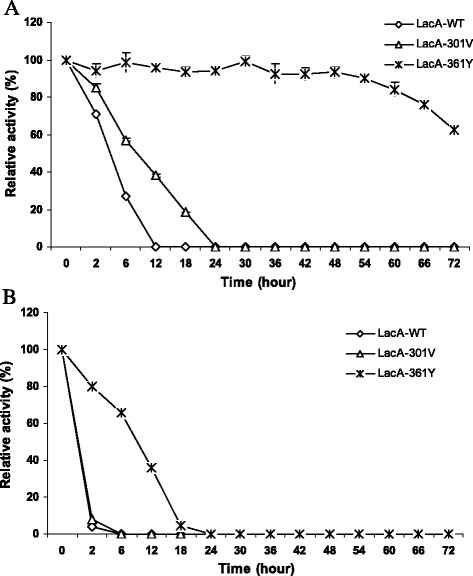
Thermostability of LacA-WT, Lac-301 V and LacA-361Y at 45 °C (**a**), and at 50 °C (**b**). The purified LacA-WT, Lac-301 V and LacA-361Y were incubated in 100 mM Na-phosphate buffer pH 7.0, and the relative activity of β-galactosidase toward oNPG (4 mg/ml) was determined in 100 mM buffer Z (pH 7.0) at 55 °C

In buffer, LacA-WT, LacA-301 V and LacA-361Y were stable in the temperature range of 30–40 °C with almost unchangeable activity after incubation for 72 h. The activity retained 95–100 % of their original activity. Interestingly, at 45–50 °C, LacA-361Y has been shown to be significantly more stable than LacA-WT and LacA-301 V (Fig. 4). LacA-361Y retained over 93 % of its original activity at 45 °C after 48 h of incubation, whereas LacA-WT and LacA-301 V lost completely after 12 and 24 h, respectively, of incubation (Fig. 4a). The half-life of LacA-361Y (83.89 ± 0.78 h) and LacA-301 V (7.6 ± 0.19 h) at 45 °C were about 26.8 and 2.4 times higher, respectively, in comparison to the half-life of LacA-WT (Table 4). At temperature optimum 50 °C, LacA-361Y has also been shown more stable than LacA-WT and LacA-301 V. LacA-WT and LacA-301 V retained only 3.72 and 7.95 %, respectively, after 2 h of incubation, whereas LacA-361Y retained 79.88 % of its original activity (Fig. 4b). At 50 °C, LacA-WT and LacA-301 V hydrolysis activity were lost completely after 6 h of incubation, whereas LacA-361Y activity was lost completely after 24 h. The half-life of LacA-361Y was higher 12.7 and 9.39 times than its of LacA-WT and LacA-301 V, respectively (Table 4). At 55–60 °C, all three enzymes have been shown to result in a complete loss of hydrolysis activity after 2 h of incubation.

**Table 4:** The half-life of wild-type and mutants LacA at various initial oNPG concentrations (w/v) and temperatures

Enzyme	Half-life (h) at various initial oNPG concentrations (w/v) and temperatures							
	45 °C				50 °C			
	0 %	5 %	10 %	15 %	0 %	5 %	10 %	15 %
WT	3.13 ± 0.14	29.36 ± 0.11	16.11 ± 0.15	11.81 ± 0.09	0.42 ± 0.03	3.75 ± 0.04	2.98 ± 0.06	1.79 ± 0.03
A301V	7.6 ± 0.19	6.52 ± 0.09	7.15 ± 0.12	6.79 ± 0.21	0.57 ± 0.03	0.81 ± 0.03	0.73 ± 0.008	0.49 ± 0.01
F361Y	83.89 ± 0.78	99 ± 0.33	90 ± 0.24	92.4 ± 0.52	5.4 ± 0.15	6.16 ± 0.23	7.1 ± 0.14	6.21 ± 0.08

The activity of β-galactosidase was determined in 100 mM buffer Z (pH 7.0) at 55 °C with substrate oNPG (4 mg/ml)

In the presence of substrate oNPG, LacA-WT was a higher thermostability than that in buffer, and was the most stable in 5 % (w/v) of oNPG solution. At 45 °C and 50 °C, the half-life of LacA-WT in presenting of 5–15 % (w/v) oNPG substrate were more stable about 3.8–9.4 times and about 4.3–8.9 times, respectively, in comparison with enzyme incubating in the buffer (Table 4). Whereas, the thermostability of LacA-301 V and LacA-361Y in the presence of substrate were not much different compared to that of enzymes incubating in the buffer.

### Characterization of mutants

The enzyme kinetics toward the oNPG substrate were compared in Table 5. The mutant enzymes LacA-301 V and LacA-361Y had a decrease in Michaelis constant (*K*_*m*_), the turnover rate (*K*_*cat*_) and catalytic efficiency (*K*_*m*_*/K*_*cat*_). *K*_*m*_ for LacA-301 V (5.57 mM) and LacA-361Y (8.02 mM) was lower than that in LacA-WT (9.28 mM) showed that the mutation of A301 to valine and F361 to tyrosine induced a decrease in the substrate affinity. The catalytic efficiency of LacA-301 V and LacA-361Y were 28.65 and 45.5 %, respectively, compared to wild-type enzyme. This result showed that A301 and F361 of LacA could be interacted with the substrate.

**Table 5:** Kinetic parameters of the purified wild-type and mutant enzymes toward oNPG as substrate

Enzyme	V_max_ (IU/mg)	K_m_ (mM)	K_cat_ (s^−1^)	K_cat_/K_m_ s^−1^mM^−1^	K_cat_/K_m_ (%)
Wild-type LacA	2.61 ± 0.12	9.28 ± 0.72	21.74 ± 0.99	2.35 ± 0.08	100
LacA-301 V	0.76 ± 0.02	5.57 ± 0.56	3.72 ± 0.09	0.67 ± 0.08	28.65
LacA-361Y	1.03 ± 0.03	8.02 ± 0.27	8.56 ± 0.24	1.07 ± 0.01	45.50

*K*_*m*_ Michaelis constant, *K*_*cat*_ turnover rate, *K*_*cat*_ */K*_*m*_ catalytic efficiency

The relative activity of β-galactosidase was determined in 100 mM buffer Z (pH 7.0) at 55 °C with oNPG as a substrate

## Discussion

### Screening and identification of random mutagenesis

The random mutagenesis method epRCA is a rapid and simple method and epRCA products are directly transformed into *E. coli* JM109(DE3). Although, the transformation efficiency was significantly higher (116 times) when transfer the epRCA products digested with *Mlu*I and ligated into *E. coli* JM109(DE3). This result was in agreement with the report that the digestion of RCA products by a single-cut restriction enzyme and self-ligation by T4 DNA ligase dramatically increased the *E. coli* XL1-blue transformation efficiency (approximately 5000 transformants per 50 ng of RCA products), whereas the direct transformation of these RCA products only resulted in a few transformants [27]. One possible explanation for this is smaller size of epRCA products after cutting by restriction enzyme *Mlu*I. Thus, the transformation efficiency of monomeric epRCA products is higher than the multimeric forms.

**Fig. 5:**
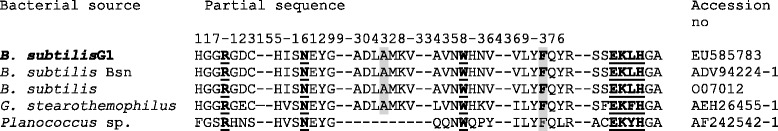
Partial sequence alignment of amino acid sequences of LacA and β-galactosidase belonging to GHF-42 from different bacterial sources. Seven positions R120, N158, W331, E371, K372, L373 and H374 predicted in interaction with substrate were shown by underline. The black bars showed two mutagenesis positions 301 and 361

The result of site-mutagenesis demonstrated that A301 and F361 play an important role maintaining activity of LacA. Using SWISS-MODEL program (http://swissmodel.expasy.org/interactive), UniProtKB/Swiss-Prot to predict model of LacA showed the active site containing E159 and E323, and the substrate binding site containing R120, N158, W331, E371, K372, L373, and H374. Therefore, the amino acids of E62V, R77W, A191V, A301V, F361 and A524 were not the active site or substrate binding site of LacA (Fig. 5). However, when it using alignment of the putative amino acid sequences of β-galactosidase belonging to GH42 revealed the F361 of LacA corresponds to F347, an active site of a cold active β-galactosidase (bgaA, AF242542-1) from *Planococcus* sp. classified as a GH42. Surprisingly, the changing phenylalanine to tyrosine at position 347 of bgaA also caused a decrease in activity from wild-type of between 40 and 50 % on oNPG substrate [26]. This suggested that F361 might interact with the substrate in hydrolysis of LacA.

### Characterization of mutants

The optimum temperature and pH of LacA-WT, LacA-361Y and LacA-301 V were obtained at the same 50–55 °C and pH 6.5 (Fig. 2). These results were coincident with the optimum temperature and pH of the β-galactosidase from *B. subtilis* KL88 [27], *Arthrobacter* sp. 32c [28].

LacA-WT, LacA-301 V and LacA-361Y were the pH stability the same at pH 5.0 to pH 9.0. Other β-galactosidases from *B. coagulans* RCS3 and *B. megaterium* 2-37-4-1 were also reported to be stable at a neutral pH range: pH 6–9 [29, 30]. However, the interesting difference in the thermostability of mutant LacA-361Y. In the buffer, at 45–50 °C, LacA-361Y shown to be significantly more stable than LacA-WT and LacA-301 V. These results might explain that the hydroxyl group of the tyrosine interacted with the carboxyl group of a certain residue, therefore, it is intended in order to to increase the structure stability. In a recent report, Dong et al., [23] also found Ile42 of BgaB belong to GH42 from *Geobacillus stearothermophilus* affected to both catalysis and thermostability simultaneously. The replacement Ile42 with polar AA enhanced the thermostability but decreased the catalytic efficiency of BgaB [23].

In the presence of substrate oNPG, LacA-WT was a higher thermostability than that in buffer, whereas LacA-301 V and LacA-361Y were not. The higher thermostability of enzyme in the presence of substrate, which might be caused complexion to the substrate or with a remaining galactose, was expected by Warmerdam et al. [31]. This may explain why the thermostability of mutant LacA-301 V and LacA-361Y did not change significantly in presence of substrate because K_m_ of mutant enzymes were lower than LacA-WT, and this result may decrease interaction of mutants LacA with substrate.

## Conclusions

Error prone rolling cycles amplification (epRCA) has been used in this study as a powerful tool to modify properties of the β-galactosidase from *B. subtilis* VTCC-DVN-12-01. These findings demonstrated the amino acids A301V and F361 play important role in hydrolysis activity of β-galactosidase from *B. subtilis*. Especially, amino acid F361 had significant effect on both catalytic and thermostability of LacA enzyme suggesting that F361 is responsible for functional requirement of the GH42 family. The finding could be applied to modify the other families of GH-42 β-galactosidase for the evolution properties of enzyme.

## Methods

### Chemicals and reagents

TempliPhi100DNA amplification kit was purchased from Roche (Basel, Switzerland). Ortho-nitrophenyl-β-D-galactopyranoside (oNPG), isopropyl thio-β-D-galactoside (IPTG), peptone, and yeast extract were provided from Bio Basic Inc. (Ontario, Canada). ProBon™nickel-chelating resin was supplied by Invitrogen Corp. (Carlsbad, CA, USA). The PCR reagents, restriction endonucleases, T4 DNA ligase and *Taq* polymerase, PCR primers(IDT, USA) were purchased from Fermentas (Thermo Fisher Scientific Inc., Waltham, USA).

### Bacterial strain and expression plasmid

The gene *lacA* (2061 bp, accession No EU585783) coding for β-galactosidase from *Bacillus subtilis* strain VTCC-DVN-12-01inserted into the expression vector pET22b(+) resulting in pELacA was described in a previous study [20]. The *Escherichia coli* strain JM109(DE3) [*end*A1, *rec*A1, *gyr*A96, *thi*, *hsd*R17 (r_k_^−^, m_k_^+^), *rel*A1, *sup*E44, λ-, ∆(*lac*-*pro*AB), [F’, *tra*D36, *pro*AB, *lac*I^q^Z∆M15], IDE3] (Promega Corp, Madison, WI) and the plasmid pELacA were used for the expression of the wild-type and mutant β-galactosidase LacA and for screening of LacA mutants. *E. coli* cells were cultivated in Luria-Bertani (LB) medium containing1% (w/v) bacto tryptone, 0.5 % (w/v) yeast extract, 1 % (w/v) NaCl, pH 7–7.5 and 50 μg/ml of ampicillin. LB agar contained additionally 2 % (w/v) agar and 100 μg ampicillin/ml.

### Error-prone rolling circle amplification

The recombinant plasmid pELacA was used as a template for the epRCA reaction by using the TempliPhi 100 DNA amplification kit consisting of a sample buffer containing random hexamers that prime DNA synthesis nonspecifically, an enzyme mix containing Φ29 DNA polymerase and a reaction buffer containing deoxyribonucleotides. An amount of 25 pg of pELacA was mixed with 5 μl of sample buffer and heated at 95 °C for 3 min to denature the plasmid, then immediately cooled down to room temperature. The amplification was started by adding 5 μl of reaction buffer, 0.2 μl of enzyme mix and MnCl_2_ at the final concentration of 1.5 mM and incubated to 30 °C for 24 h. The mixture was heated at 65 °C for 10 min to inactivate the enzyme. The quantity of amplified DNA was estimated on 1 % (w/v) agarose gel electrophoresis and by measuring its absorbance at 260 nm with a spectrophotometer UV-2500 (LaboMed Inc., Culver City, CA, USA).

### Construction of mutant libraries

epRCA products were digested with *Mlu*I in a mixture contained 12 μl (600 ng) of ep-RCA product, 5 μl 10 × buffer R, 2 μl (20 U) *Mlu*I and 31 μl H_2_O. After 6 h of incubation at 37 °C, the digested products were purified by using the MinElute Reaction Cleanup kit (Qiagen) and ligated with T4 ligase. The ligated epRCA transformed into *E. coli* JM109(DE3) by using standard electroporation method with a 0.1 cm electrode cuvette under the conditions at 1.8 kV, 200 Ω, and 25 F. Transformed cells were plated onto LB plates containing 50 μg/ml of ampicillin and incubated at 37 °C overnight. Colonies harboring putative mutation sites in *lacA* were prescreened for a higher β-galactosidase production.

### DNA manipulations

Plasmid DNA isolation was carried out by methods as previously described [20]. DNA sequencing was performed on ABI PRISM 3100 Avant Genetic Analyzer (Applied Biosystems Inc., Foster City, USA). Sequence alignments constructed and analyzed using the program MegAlign DNAStar. *E. coli* DH5α and JM109(DE3) cells were transformed using heat shock method that has been previously described [20].

### Screening β-galactosidase activity of mutants

To screen β-galactosidase activity of mutants, the individual transformants carrying the putative mutant *lacA* gene were randomly selected and grown in 300 μl LB medium containing 100 μg/ml ampicillin in 96-deep well plates at 37 °C overnight with agitation of 250 rpm. 25 μl of overnight culture was transferred from each well to second 96-deep well plates containing 300 μl LB medium containing ampicillin. The culture was cultivated at 37 °C with agitation of 300 rpm until an optical density (OD) at 600 nm of 0.6 to 0.8 was reached (for approximately 4 h), then 1 mM isopropyl-β-D-thiogalactopyranoside (IPTG) was added. The culture continuously incubated at 37 °C with agitation of 300 rpm for 16 h of induction. The cell cultures were used as the enzyme source to screen the activity.

The procedure for measuring β-galactosidase activity of colonies from site-saturation library was performed according to Griffith et al., [32] with oNPG substrate (4 mg/ml) in 0.1 M Na-phosphate buffer, pH 7. The absorbance of culture density at 600 nm, reaction mixture at 420 and 550 nm was measured in a microplate reader Elx800™ (BioTek Instruments Inc., Winooski, USA) and β-galactosidase activities were calculate in Miller units following equation: Miller Unit (*nM*/min/*OD*_*cell*_) = [(*OD*_420_ − 1.75 × *OD*_550_)] × *V*_1_/(*T* × *V*_2_ × *OD*_600_). In that, OD_420_ and OD_550_ are read from the reaction mixture; OD_420_–1.75 × OD_550_, nmoles formed per milliliter; 1.75 × OD_550_, light scattering at 420 nm; T, incubation time (min); V_1_ (ml), total assay volume; V_2_ (ml), volume of culture used in the assay; OD_600_ reflects cell density in the washed cell suspension. All measurements were carried out in triplicate with the resulting values being the mean of the cumulative data obtained.

### Site-directed and saturation mutagenesis

Site-directed and saturation mutagenesis were performed by a one-step polymerase chain reaction (PCR) method, using plasmid pELacA as template and a pair of mutagenic primer. Randomization codon was performed with a pair of primer that introduced a codon NNK at selected positions. Whole pELacA plasmid was amplified in PCR mix containing 5 μl of 10× PCR buffer, 4 μl of 25 mM MgSO_4_, 4 μl of 2.5 mM dNTP, 0.5 μl of 2.5 U/μl *Pfu* polymerase (Thermo), 1 μl of each primer (10 pmol), 1 μl of pELacA (50 ng), and 33.5 μl H_2_O. The thermocycler conditions were performed as follow: 95 °C/4′; 18 cycles of 95 °C/30″, 54 °C/1′, 72 °C/8′; and 72 °C/10′. Then, 10 U of *Dpn*I restriction enzyme (Thermo) was added the reaction products and incubated for 2 h at 37 °C to digest pELacA template. The PCR products were purified using a PCR purification kit (Thermo). The resultant plasmid DNA was transformed into chemically competent JM109(DE3) cells. Transformants were selected on LB agar containing 100 μg/ml after incubation overnight at 37 °C.

### Enzyme expression

The transformants *E. coli* JM109(DE3)/pELacA harboring the wild type and mutant *lacA* gene were cultivated in 5 ml of LB medium containing 5 μl of 100 mg ampicillin/ml at 37 °C with agitation at 220 rpm. Five hundred μl of the overnight culture were transferred into 50 ml of LB medium containing 50 μl of 100 mg ampicillin/ml in a 250-ml Erlenmeyer flask. The culture was cultivated at 37 °C with agitation at 200 rpm until an optical density (OD) at 600 nm of 0.6–0.8 was reached (for approximately 4 h), then 50 μl of 100 mM IPTG was added. The culture was continuously incubated at 37 °C with agitation of 220 rpm for 6 h of induction. Cells were harvested by centrifugation at 5000 rpm for 10 min at 4 °C. Wet cells were used for enzyme purification.

### Enzyme purification

The recombinant LacA fused with a C-terminal 6 × histidine-tag was purified using affinity chromatography with Ni^2+^-ProBond™ resin under native conditions. An amount of 500 mg wet cells from a 50-ml culture in LB medium was harvested by centrifugation at 4000 rpm and 4 °C for 10 min, washed with 8 ml of water and resuspended in 8 ml of 1× native purification buffer containing 50 mM NaH_2_PO_4_, 0.5 mM NaCl,100 mM imidazol, pH 8.0. To the mixture, lysozyme was added at a final concentration 0.5 mg/ml and incubated on ice bath for 30 min. The cell mixture was disintegrated by ultrasonic waves (3× 1 min with 1 min pause). The supernatant of the cell lysate was obtained by centrifugation at 13,000 rpm for 10 min and loaded on to a column containing 2 ml resin, which was equilibrated with native binding buffer and incubated for 45 min at room temperature with gentle hand shaking for several times. The column was washed with 3 times of 8 ml native wash buffer. The bound protein was eluted with 8 ml of 1× native purification buffer containing 250 mM imidazol. The enzyme solution was used for characterization.

### Electrophoresis analysis and protein concentration

The homogeneity and molecular mass of the β-galactosidase was determined by 12.5 % SDS polyacrylamide gel electrophoresis [33] with Biometra equipment (Göttingen, Germany). Proteins were visualized by staining with 0.1 % (w/v) Coomassie Brilliant Blue R-250. Protein concentrations were estimated by the method of Bradford with the bovine serum albumin as standard [34].

### β-Galactosidase activity assay

To estimate the activity of the purified β-galactosidase, 1 μl (3.4 μg) purified enzyme solution was added to 74 μl 22 mM oNPG in 100 mM buffer Z (40 mM Na_2_HPO_4_.7H_2_O, 60 mM NaH_2_PO_4_.H_2_O, 10 mM KCl, 1 mM MgSO_4_.7H_2_O, 50 mM 2-Mercaptoethanol) pH 7, incubated at 50 °C for 10 min. Then the reaction was stopped by addition of 25 μl 1 M Na_2_CO_3_. The absorbance was read at 420 nm against a blank containing oNPG, buffer Z but without enzyme solution. The following equation was used to calculate units of β-galactosidase activity: *U*/*ml* = [*OD*_420_ × *V*_1_]/[0.0045 × 1000 × *T* × *V*_2_] (ii). In the equation (ii), OD_420_/0.0045×1000: μmoles what formed per milliliter; T, incubation time (min); V_1_ (ml), total assay volume; V_2_ (ml), enzyme volume used in the assay.

### pH and temperature dependency of mutant enzymes

The temperature and pH optimum of thepurified wild-type and mutant LacA, 3.4 μg enzyme for each reaction, were determined by measuring the activity as described above using 100 mM buffer Z (pH 7) at the temperature range of 30 to 70 °C and using different buffer pH 4.0–9.0 (100 mM potassium acetate buffer pH 4.0–6.0, 100 mM Na-phosphate buffer pH 6.0–8.0, or 100 mM Tris–HCl buffer pH 8.0–9.0) at 50 °C, respectively, for 5 min.

For the determination of temperature and pH stability, purified enzyme, 3.4 μg protein for each reaction, was incubated in 100 mM buffer Z at different temperatures from 30 to 60 °C for 0–72 h, and in 100 mM Na-phosphate buffer pH 4.0 and 9.0 at 30 °C for 0–72 h, respectively. The remaining activity was then determined. Half-life (T_1/2_) of each mutant enzyme was determined as follows: T_1/2_ = ln2/k, where U_t_, U_0_, and k are enzyme activity at t min, initial enzyme activity, and the apparent rate constant, respectively.

All data were averaged and experiments were performed three times. Specific activities were calculated from these averages. The entire assay experiments were than repeat two more times and three specific activity values were averaged. The errors (SD) were calculated by STDEV function of Excel.

### Characterization of mutants

The apparent kinetic parameters (V_max_ and K_m_) were determined against 0.1-6 mg/ml of oNPG as a substrate using Lineweaver-Burk plots. Activities were recorded at 55 °C and calculated on the basis of an extinction coefficient for *o*-nitrophenol of 4500 M^−1^ cm^−1^ at 420 nm.

### DNA and amino acid sequence analysis

Homologies of the DNA and amino acid sequences were determined with the program Megalign DNAStar.

## Abbreviations

Gene *lacA*, gene encoding β-galactosidase from *Bacillus subtilis* strain VTCC-DVN-12-01; Protein/Enzyme LacA, β-galactosidase from *Bacillus subtilis* strain VTCC-DVN-12-01
